# Chartered Accountants’ perception of the Fourth Industrial Revolution

**DOI:** 10.3389/fpsyg.2024.1419766

**Published:** 2024-10-17

**Authors:** Chené Brands, Claude-Hélène Mayer, Rudolf M. Oosthuizen

**Affiliations:** ^1^Department of Industrial Psychology and People Management, College of Business and Economics, University of Johannesburg, Johannesburg, South Africa; ^2^Department of Industrial and Organisational Psychology, University of South Africa, Pretoria, South Africa

**Keywords:** Chartered Accountants (CAs), Fourth Industrial Revolution (4IR), automation, acceptance, adoption, technology, perception

## Abstract

The Fourth Industrial Revolution (4IR) is an era of enormous technical progress that has impacted professionals across industries, including Chartered Accountants (CAs). This study explored how CAs view the impact of the 4IR on their profession, focusing on the shifting roles, competencies, and challenges they face during this transitional period. The research adopted a qualitative approach to data collecting, including 14 semi-structured interviews with participants from various CA backgrounds. This research provides a thorough knowledge of the 4IR’s consequences for the profession and the perceptions of CA’s of the 4IR. Rapid task automation via technologies such as AI is posing a challenge to traditional CA roles, forcing a change towards more analytical thinking and strategic insight. CAs need to develop critical thinking abilities and data analysis ability. Older generations might need support to adapt to the technological changes. Despite fears about job loss due to technology, members were largely optimistic about the 4IR’s professional development potential. Conclusions are drawn and recommendations are given.

## 1 Introduction

The 4IR is changing the world of work by combining technology and introducing significant changes to organizational operations (Bughin et al., 2018; SAICA, 2023a, 2025; Schwab, 2016). This transformation has the potential to reshape the accounting profession, influencing the responsibilities and skills needed by CAs (Kroon et al., 2021; Marx et al., 2020). CAs are projected to migrate away from manual operations and towards higher-value duties such as data analysis (AICPA and CanadaCPA, 2020). However, security concerns and technological compatibility can prevent CAs from adopting new technologies (Davies, 2022; Jackson et al., 2022). Individual perceptions influence technological acceptance (Damerji and Salimi, 2021; Ho et al., 2017; Igbaria et al., 1994). Adoption and acceptability are critical in the context of the 4IR (Cambridge, 2023a,b,c; Teo, 2011; Renaud and Van Biljon, 2008; Carr, 1999). The 4IR introduces smart technologies and automation, which challenge traditional job assumptions and require new skills (Schwab, 2016; Manyika et al., 2017; Rotatori et al., 2021). The CA profession is evolving as a result of technical improvements and digital demands (IFAC, 2017, 2023; du Chenne, 2023). CAs must adapt to remain competitive and relevant, which necessitates lifelong learning and the acquisition of new skills (SAICA, 2021, 2025; Tsiligiris and Bowyer, 2021). Adoption of IT activities in accounting can increase worker efficiency and understanding client needs (Pan and Seow, 2016; Tysiac, 2015; Hwang et al., 2016). 4IR technology such as AI, blockchain, and IoT are impacting the CA profession (Davis, 2016; Philbeck and Davis, 2018; Vandenberg, 2020). These technologies present possible opportunities and difficulties for CAs, urging skill development and adaptability (Prisecaru, 2016; Schwab, 2017; Xu et al., 2018). Understanding CAs’ perceptions and adoption of 4IR technologies is critical for company profitability and relationships with clients (Xu et al., 2018; Tsiligiris and Bowyer, 2021).

## 2 The Fourth Industrial Revolution

In 4IR, new technologies are uniting the physical, digital, and biological domains to create a new world of work (David et al., 2022; Davis, 2016; Schwab, 2017), changing and affecting the way people live, work, and interact (Schwab, 2015; Xu et al., 2018). Different professions are affected by the 4IR in numerous, positive and negative ways (Hattingh, 2018; Partridge and Brinich, 2023), resulting in shifts in power, wealth, and knowledge. 4IR brings impact of new technologies on human development (Philbeck and Davis, 2018). The 4IR is not yet clear in what way it will unfold. Schwab (2015) however emphasised the need for an integrated and comprehensive response, involving both public and private stakeholders, as well as civil society and academia. According to Professor Klaus Schwab, the founder and executive chairman of the World Economic Forum, the 4IR is characterized by several megatrends, including the Internet of Things, 3D printing, nanotechnology, blockchain, and artificial intelligence (Davis, 2016; Schwab, 2016; Vandenberg, 2020).

In this context, “smart” refers to digital rather than physical transformations (Chou, 2018). Industry 4.0 is often used interchangeably with 4IR, but these terms describe different concepts, despite their similarities. Industry 4.0, a component of the 4IR, is an initiative that originated in Germany between 2011 and 2015 (Philbeck and Davis, 2018). Manufacturing and production systems are examined with respect to the interaction between digitalization, organizational transformation, and productivity enhancement. Change driven by technology is not what the 4IR is about; it is about affecting industries’ cores in a positive way (Schwab, 2015). It is fundamentally characterized by significant changes in the creation, exchange, and distribution of economic, political, and social values (Philbeck and Davis, 2018). In almost every industry, the 4IR is evolving exponentially (Schwab, 2015). The result is a change in the entire production, management, and governance system (Schwab, 2015). As stated by Coman et al. (2022), AI will automate the routine and repetitive tasks of CAs. CA professionals will still require human intellect. Gonçalves et al. (2022) also affirmed that analysis, exploration, synthesis, and interpretation of data are the most valuable activities of a *CA*. The CAs will need to develop new skills as they move from operational-based initiatives to more strategic initiatives (Domil et al., 2022; Awang et al., 2022).

Humans and technology interact in new ways as a result of industrial revolutions in the systems surrounding them (Philbeck and Davis, 2018). Therefore, technology plays a crucial role in how people perceive the world and interact with each other (Almeida et al., 2020; Davis, 2016; Philbeck and Davis, 2018; Turner, 2022). Technology perception is often influenced by the complexity of these technologies and their unfamiliarity and threat (Ahn and Chen, 2022; Davis, 2016; Philbeck and Davis, 2018; Zervoudi, 2020). Technological advancements are allowing individuals to benefit significantly from these new technologies (Khurana et al., 2022; Pirhonen et al., 2020; Voelpel et al., 2005).

### 2.1 The Fourth Industrial Revolution and its impact on Chartered Accountants

The CA profession consists of high-performing, forward-thinking professionals who understand financial, technical, and operational concepts (SAICA, 2023b, 2025). To shape the strategic direction of organizations, CAs use their expertise in financial analysis and business insight (SAICA, 2023b). To achieve this, CAs act as business guides by measuring, assessing, and reporting on companies’ sustainable development (Andreev et al., 2022; Grosu et al., 2023; Sitaram et al., 2022). A new generation of digital accounting platforms is driving the CA profession to provide new services to clients (Grosu et al., 2023), such as Blockchain, big data, cloud computing, and artificial intelligence, which will make large-scale decision-making more automated, allowing CA professionals to perform more complex tasks (Grosu et al., 2023).

Prior to the advent of 4IR, the accounting profession was governed by extensive technology (Chaplin, 2017). Workplace integration of 4IR technologies has an unpredicted impact on the world of work (Faizal et al., 2022). Sectors and industries will influence the future world of work in various ways, including role disruption, financial expenditures for adopting new technologies, skills, knowledge, and access, as well as employee adaptability (Abe et al., 2021; Tsiligiris and Bowyer, 2021). It is therefore necessary for the CA profession to diversify its ability to adopt these new digital technologies (Faizal et al., 2022). Using these technologies will enable CAs to support data-driven organizations (Chu and Yong, 2021; Grosu et al., 2023; Ibrahim et al., 2021). In addition to automating repetitive and mundane tasks, these technologies also increase managers’ access to information, thus enhancing decision-making (Awang et al., 2022; Balios, 2021; Grosu et al., 2023).

Furthermore, professional accounting bodies align their competency frameworks with the skills needed to function in the 4IR (Barac et al., 2021; du Chenne, 2023). Consequently, professional bodies, academia, and training must contribute to this transformation and equip CAs with the necessary skills and knowledge, which may be a challenge. In addition to the International Auditing and Assurance Standards Board (IAASB, 2023), the International Ethics Standards Board for Accountants (IESBA, 2023), the International Education Standards Board (IAESB), and the International Public Sector Accounting Standards Board (IPSASB) set international standards.

### 2.2 Perception of Fourth Industrial Revolution technologies

Industrial and organizational psychology has extensively researched perception (Farid et al., 2019; Guo et al., 2020; Rudolph et al., 2021; Weber et al., 2020). Organizations and their employees are affected by perception in numerous ways (Farid et al., 2019; Guo et al., 2020; Rudolph et al., 2021; Weber et al., 2020). It is important for industrial and organizational psychologists to understand the implications of these findings. What is perception, and how does it affect behaviour? Perception, according to Gibbs (2005), is the ability to interpret sensory input. According to Ittelson and Kilpatrick (1951), people act in accordance with what they perceive, leading to new behaviours. Therefore, actions are continuously influenced by perceptions and perceptions are continuously influenced by actions (Ittelson and Kilpatrick, 1951).

One of the most controversial fields in psychology is understanding how and why individuals perceive (Ittelson and Kilpatrick, 1951). Perception can be influenced by several factors, including prior experiences, cultural background, and individual biases. To understand how people perceive and respond to their environment, it’s crucial to study perception (Bickerstaff, 2004). Multiple responses can be elicited by a straightforward stimulus, according to Perugini and Prestwich (2007). As a result, perception involves the interpretation and understanding of sensory information (Chalmers et al., 1992; Zha et al., 2022). Several empirical studies and theories have demonstrated its influence on human behaviour (Dillon and Morris, 1996; Lee, 2009; Lim and Zhang, 2022; Luo et al., 2010; Pavlou, 2003; Rauniar et al., 2014).

Individual perceptions influence attitudes, beliefs, and decision-making processes (Meijer et al., 2015; Venkatesh et al., 2000). It affects how people perceive and interpret the world, as well as their willingness to accept new ideas or change their behaviours (Lippert and Davis, 2006; Tu and Hu, 2018). According to Grosu et al. (2023) study on CAs perceived digital readiness, CAs perceptions of the need for adoption and perceived importance and benefits will determine their behaviour towards learning new skills and knowledge. Further, the perceived threat of job or role redundancy would influence CAs behaviour (Grosu et al., 2023). Venkatesh et al. (2003) explains how people adopt and perceive technology by using the UTAUT model (Venkatesh et al., 2003). Four core determinants of technology adoption are identified by combining elements from a variety of technology acceptance theories: performance expectancy (how it enhances performance) (Zhu et al., 2010; Venkatesh et al., 2016), effort expectancy (ease of use), social influence (others’ opinions), and facilitating conditions (support infrastructure) (Venkatesh et al., 2003). In addition to gender, age, experience, and voluntariness of use, UTAUT considers gender, age, and experience. Adoption and acceptance of technology are guided by this theory.

## 3 Research methodology

### 3.1 Research paradigm and strategy

This study utilised a qualitative research approach to study CAs perceptions in an international organizational environment (Tenny et al., 2023). The goal of qualitative research, a non-experimental approach, is to explore CAs experiences, perceptions, and behaviours in-depth (Pathak et al., 2013; Tenny et al., 2023). Semi-structured interviews were conducted to accomplish this, in accordance with Denzin and Lincoln (2008) concept of qualitative research as a naturalistic, interpretative method that investigates events in their natural environment, concentrating on the meaning individuals assign to these phenomena.

The study used Dilthey (1976, 2002) modern hermeneutics as a research paradigm to contribute to the “Verstehen” (engl. Understanding) of the perceptions described by the sample. At the same time, the researchers applied a self-reflective attitude towards the interpretation of the perceptions of the narrators in the study (Ratner, 2002; George, 2020).

In terms of the research strategy, key characteristics of qualitative study include the exploration of hypotheses, the use of semi-structured methodologies for data collection, flexibility to capture diverse perspectives, the use of deductive reasoning, and the appreciation of contextual variables (Flyvbjerg, 2006; Kim et al., 2017; Nassaji, 2020). This method enables the researchers to delve deeply into participants’ opinions, providing a thorough comprehension of the research topic.

### 3.2 Study population and sample

The study population included CAs from various international firms who were registered as CAs or CA clerks with the relevant regulatory boards. Participants had to be over the age of 18 and fluent in English, Afrikaans, or Dutch, as well as work for an organisation as a CA or CA clerk. The researchers selected 14 individuals using non-probability selection approaches, specifically purposive and convenience sampling (Berndt, 2020; Etikan and Bala, 2017). Purposive sampling guaranteed that participants met inclusion criteria, whereas convenience sampling made it possible to pick accessible and willing individuals (Campbell et al., 2020; Etikan et al., 2016; Obilor, 2023). Participants were contacted via email or LinkedIn, and data collection continued until data saturation was reached (Braun and Clarke, 2021; Morse, 1995; Saunders et al., 2018). This ensured that a thorough grasp of the participants’ perspectives and experiences was gained. The sample size of 14 participants was diverse, representing various countries, backgrounds, age categories, and nationalities (Berndt, 2020). The researchers acquired access to participants by presenting the goal of the study and requesting them to willingly participate in one-on-one online interviews (Etikan and Bala, 2017). The sample and overview of the participants included individuals from 6 different countries and between 24 and 44 years. Altogether, 14 participants participated, four were female and ten were male.

### 3.3 Data collection and analysis

This study used online semi-structured interviews through MS Teams, which is a typical method in qualitative research (Taylor, 2005; Dicicco-Bloom and Crabtree, 2006). Researchers asked questions 10–15 questions, such as “What is your perception of the potential impact of 4IR technologies on the chartered accounting (CA) profession?”; “What do you perceive to be potential challenges?”; and “Where do you see the profession in the next 15–20 years in the 4IR – what will have changed by then?” The participants stated that they were fine with using MS Teams as an interview platform.

Data collection was stopped when data saturation was reached. This means that the researchers collected all needed and relevant information and that no newer information could be obtained and that the researcher could fulfill research questions and objectives through the collected data (Fusch and Ness, 2015).

Thematic analysis was utilised to detect reoccurring themes (Campbell et al., 2021), giving a comprehensive picture of participants’ responses (Alshenqeeti, 2014). The interviews included open-ended questions with no time constraints but a recommended duration of 30–60 min was kept in mind (Guetterman, 2015). Recordings were accurately transcribed using MS Teams’ record and transcribe feature (Alshenqeeti, 2014; Braun and Clarke, 2021). The researcher confirmed data saturation, corrected transcribing errors, and eliminated personally identifiable information and redundant words (Surmiak, 2018). Reflexivity was utilised to eliminate research bias (Dodgson, 2019), contributing to the rigor and reliability of the data analysis process. Reflexivity was applied by reflections about research findings in the light of the theory, as well as through discussions of the researchers.

Thematic analysis is widely employed in qualitative research to detect patterns and themes to understand the topic of research in-depth (Boyatzis, 1998; Braun and Clarke, 2006). This study used Braun and Clarke’s six-step approach in conjunction with ATLAS.ti software (Alhojailan, 2012; Maguire and Delahunt, 2017). The steps included becoming acquainted with the data (the researchers read through the datasets and explored them), creating codes, spotting patterns, reviewing and defining themes (data were arranged, rearranged, and discussed), and synthesising findings (Braun and Clarke, 2006). ATLAS.ti was used to produce codes, with a focus on frequent codes to inform theme generation while avoiding preconceived frameworks (Nowell et al., 2017). Inductive reasoning guided the analysis to gain an accurate understanding of perceptions (Nowell et al., 2017). Transcripts with parallel themes included additional remarks and interpretations (Nowell et al., 2017). Data confidentiality was ensured by storing the transcripts on a password protected computer in a password protected file (Alshenqeeti, 2014).

### 3.4 Qualitative quality criteria

Quality was upheld in this study by thorough planning, execution, and transparency to ensure that the research can be replicated and is free of biases (Johnson et al., 2020). To preserve rigor, the researchers undertook a clear and systematic data analysis using multiple sources. Trustworthiness was maintained by rigorous record-keeping and participant debriefing for clarity and validation (Nowell et al., 2017; Thomas, 2017). Transferability was addressed by providing detailed descriptions and definitions, which facilitated replication and application in a variety of situations (Lincoln and Guba, 1985). Final transcripts were provided to participants for accuracy verification, ensuring that their experiences were accurately described, thereby improving study credibility and transferability.

### 3.5 Ethical considerations

Ethical principles such as non-malfeasance, voluntary participation, informed consent, privacy, and confidentiality guided this study (De Vos et al., 2011; Munhall, 1988; White, 2020; World Health Organization, 2020). Participants were protected from harm, received clear details of the study, and signed consent papers providing them their rights as research participants (Neuman, 2011). Debriefing sessions following interviews fostered transparency and respect for participants (Neuman, 2011). Interview recordings were securely archived, and questioning tactics ensured participants’ dignity and confidentiality (Cychosz et al., 2020; Blanche et al., 2006).

## 4 Findings

**Figure 1:**
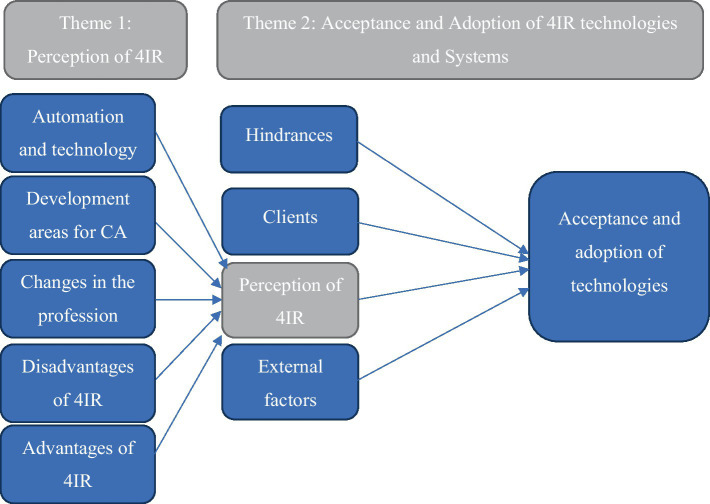
Chartered Accountants’ perceptions and acceptance and adoption of Fourth Industrial Revolution technologies.

Figure 1 presents the overview of CAs perceptions of the 4IR, as well as their acceptance and adoption of 4IR technologies, and how these two themes interact. Four categories were discovered within the theme of CAs perception of the 4IR. The four categories, namely technological automation, CAs development areas, 4IR advantages, and 4IR disadvantages, all have an impact on CAs perceptions of the 4IR. This then leads to theme two: acceptance and adoption of 4IR technology. According to the findings, the acceptance and implementation of 4IR technologies are influenced by five categories: hindrances related with the acceptance and adoption of these technologies, clients, and the CAs perception (theme one), external factors and CAs knowledge about or knowledge expectancy to adopt these technologies.

### 4.1 Chartered Accountants’ perception of the Fourth Industrial Revolution

**Figure 2:**
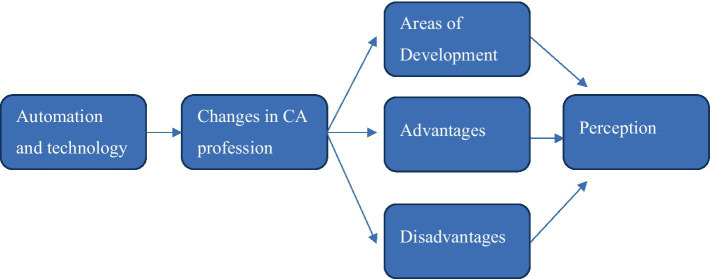
Chartered Accountants’ perception of the Fourth Industrial Revolution.

Figure 2 depicts the findings of CAs perception of the 4IR and how the various elements linked to one another. CAs perceived the 4IR as the automation of several jobs and positions, which resulted in changes to the profession. These changes influenced the CAs perceptions of areas where they needed to improve or upskill (development areas), the benefits of task and role automation, and the perceived downsides of these changes. All these elements influenced and shaped the CAs view of the 4IR.

The participants’ impressions of the 4IR differed greatly, indicating different perceptions on its impact on the accounting profession. Some participants welcomed the automation and technology developments brought forth by the 4IR, seeing them as an opportunity to improve efficiency and service quality.

Automation is reshaping the CA profession, particularly in the context of auditing (all participants). As P1 mentioned:

You could definitely identify those and then just focus on how do we think through and verify those things as opposed to deploying a massive team… you automate the whole thing and provide assurance using AI.

Participant 7 shared this opinion, saying,

The integration of AI and machine learning has significantly improved our ability to provide timely and accurate financial insights to clients.

Others, however, expressed concerns about the possible disruptions, job displacement, and privacy risks that come with growing digitization. Participant 12 expressed these worries, adding,

While technology brings many benefits, we also need to address the challenges it poses, such as data privacy and the need for upskilling to remain competitive.

Participants emphasised the advantages of incorporating AI and machine learning into accounting procedures, which streamline mundane activities and enable a move to higher-value advising services. Participant 3 emphasised this, saying,

Automation has freed up time for us to focus on strategic financial planning, adding value to our clients’ businesses.

The conversation on changes in the CA profession was a mix of optimism and trepidation. While some participants welcomed new roles and responsibilities as a result of technology integration, Participant 5 expressed concern, stating,

The evolving landscape requires us to adapt quickly, but it also brings uncertainties regarding job roles and skill requirements.

P2 highlighted the expectation of new roles and expanded training, indicating that CAs must be better prepared for the forthcoming changes in practice:

They are going to be encouraged to take up new roles, and I think the training that we’re going to get is probably going to expand to incorporate more of the 4IR aspects so that Chartered Accountants are more prepared for the change that they will see in practice.

Continuous learning and upskilling have emerged as critical areas for CAs to stay relevant in a fast-changing digital economy. Participant 9 emphasised the significance of this, adding,

Continuous learning is essential to keep pace with technological changes and deliver value-added services to clients.

P3 noted:

If they can implement that into the curriculum or the competency basis, I think that would improve Chartered Accountants or their training period definitely because when I was doing my articles, I think what lacked quite a bit was the technology aspects.

Privacy problems, legal challenges, and probable job redundancies were identified as downsides of the 4IR. However, members recognised the benefits, which included increased efficiency, better client service, and chances for professional development and innovation. Participant 11 emphasised this, stating,

While there are challenges, the 4IR also presents opportunities for us to enhance our services and expertise.

### 4.2 Acceptance and adoption of Fourth Industrial Revolution technologies

**Figure 3:**
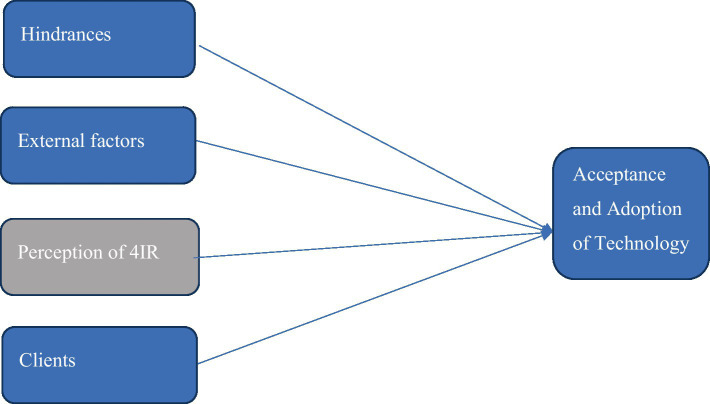
Chartered Accountants’ acceptance and adoption of Fourth Industrial Revolution technologies.

Figure 3 illustrates the factors influencing CAs adoption and acceptance of 4IR technology according to the participants’ responses. The factors are their perception of the 4IR, as mentioned previously, and hindrances, external factors and clients. These factors all contribute to CAs willingness to accept these technologies in practice and adopt them in their day-to-day working lives.

Regulatory restrictions, organizational preparation, market dynamics, and client expectations all had a role in CAs acceptance and use of 4IR technology. Although these factors are interchangeable and influence one another for the findings, it is important to highlight each separately as they relate to different perspectives.

#### 4.2.1 Hindrances

P2 highlighted the rapid pace of technological change that could lead to CAs feeling overwhelmed and uncertain in the face of these changes (P1, P2, P4, P5, P7, P8, P9, P10, P11, P12, P13, P14):

There’s always something new that’s coming out. So it might feel a little bit overwhelming for you to keep up with all the change that’s happening.

Participants cited legislative difficulties, organizational opposition to change, and talent shortfalls as major barriers to technology adoption. Participant 8 stated his concern, saying:

Navigating regulatory requirements and upskilling our workforce are critical challenges in adopting new technologies.

Due to the technology and trends changing often, CAs could feel uncertain about adopting and accepting technologies and technological systems. This is because it requires time and effort from the CA to learn about and adopt new technologies. The fear is that by the time the CA have mastered one system, another might be trending and, therefore, be implemented. This could lead to fear of using technology that is being implemented that could lead to a decreased willingness to adopt and accept the technology (P1, P2, P3, P4, P5, P6, P7, P10, P11, P12, P13, P14). As P1 pointed out: *“The ‘black boxness’ is a real challenge and very scary.”*

#### 4.2.2 External factors

External reasons including market competitiveness, customer need for real-time reporting, and industry technological breakthroughs all helped to drive technology adoption among CAs. Participant 6 emphasised this by stating,

Client expectations and industry trends play a significant role in shaping our technology adoption strategies.

CAs adoption and acceptance of 4IR technologies were influenced by the interplay of numerous external factors, namely, social factors (P1, P2, P4, P5, P6, P8, P10, P11, P12) as well as economic background and infrastructure (P2, P5, P9, P12). These overlapping codes are evident in various perspectives of the CA participants. Social factors refer to social media (P1, P4, P5) and social interactions and exposure (P1, P2, P4, P8), playing an important role. As mentioned by P1:

There are different levels of awareness of 4IR and what it entails across different parts of society.

P1 illustrates how an individual’s background and exposure to the 4IR could influence their willingness to accept and adopt the technologies that come with the 4IR. Awareness was also affected by social media and access to social media (P4, P5). As P4 noted:

The world is run by social media and what everyone says and promotes online.

This highlights the role of social factors and social media in the impact on acceptance and adoption of technologies. Therefore, P1’s quote refers to different levels of awareness depending on societies, indicating that a person’s exposure could depend on their social background.

#### 4.2.3 Clients

Clients’ increased reliance on technology-driven solutions, as well as their level of digital maturity, influenced CAs technology adoption decisions significantly. Participant 4 emphasised the importance of this, noting,

Understanding our clients’ digital needs and aligning our services accordingly is essential for staying competitive in the market.

If clients are unwilling to implement new strategies and expect their CAs to be up to date with the new trends in terms of technologies, it hinders the CA’s ability to adopt the systems and processes. P12 reiterated this challenge as he stated:

I just like audited a big company in Europe a few weeks ago, they turned like 400 million profit a year. But they don't want to pay to transition over to like a cloud computing system, because they stuck in the “old and why spend this money if it's worked before”.

In summary, various aspects profoundly affect CA’s pace of adoption and acceptance of 4IR technologies. The fear of rapid technological change, job redundancy, social aspects, economic background and infrastructure creates a complex landscape in the CA profession. Additionally, CA’s clients’ readiness to accept and adopt these technologies plays a crucial role in the rate of acceptance and adoption for the *CA*. Therefore, understanding and resolving these obstacles will be crucial to ensuring a seamless transition and successful integration of these transformational technologies as the accounting profession continues to develop within the framework of the 4IR.

## 5 Discussion

The CAs views of the 4IR are consistent with prior research, emphasising both opportunities and challenges. The optimistic attitude on automation and technology is shared by Bughin et al. (2018) and Schwab (2016), who emphasise the revolutionary power of AI and machine learning in improving business processes. However, concerns about employment displacement and privacy issues reflect the warnings issued by Marx et al. (2020) and Manyika et al. (2017) on the societal consequences of rapid technology breakthroughs.

The findings on the influence of the 4IR on the CA profession contribute to the current debate in the literature. The trend to higher-value advisory services is consistent with the evolution highlighted by the SAICA (2025), AICPA and CanadaCPA (2020), and Jackson et al. (2022), which emphasises the need for CAs to expand beyond traditional duties. The concerns raised about work responsibilities and requirements for skills are consistent with the findings made by IFAC (2017) and Teo (2011) about the importance of ongoing upskilling in response to technological disruptions.

The identified development areas for CAs are consistent with prior literature that emphasises the significance of continual learning and skill development in the 4IR era (Davies, 2022; Renaud and Van Biljon, 2008). The emphasis on upskilling to remain competitive is consistent with the recommendations made by Tsiligiris and Bowyer (2021) and Hwang et al. (2016) for professional adaptation to technological developments. The barriers to technology adoption mentioned by participants are consistent with earlier findings (Barac et al., 2021; Xu et al., 2018). Regulatory complexity and organizational resistance to change are common themes in the literature (Curtis et al., 2009; Damerji and Salimi, 2021), emphasising the ongoing problems of integrating new technologies into accounting processes.

Overall, the findings from the CAs perception give useful insights that supplement and extend current literature on the effects of the 4IR on the accounting profession, as well as CAs growth goals and issues with technology adoption. These findings contribute to a more nuanced understanding of the changing function of CAs in the digital era, emphasising the need of strategic adaptation and ongoing learning in navigating technological revolutions.

CAs may feel unsure about when to adapt and accept new technologies due to the rapidly changing nature of technology and trends. Participants (P1, P2, P4, P5, P7, P8, P9, P10, P11, P12, P13, P14) voiced concerns about the changing nature of technology, believing that the time and effort expended on learning one system would be rendered useless when other trends develop. This corresponded with the emphasis in the literature on the 4IR’s rapid technical evolution (Schwab, 2016). The current study extended the findings of previous literature by highlighting how CAs may perceive the need to continuously adapt to evolving technology trends, leading to uncertainty about the optimal timing of adoption.

The “*black boxness*” as a challenge and a cause of anxiety, as described by P1, highlighted how important it is to comprehend technology to reduce ambiguity and resistance.

Lastly, generational differences could affect the acceptance and adoption of technology and the perceived benefits and challenges of using and implementing the technology in the CA profession (P5, P6, P7, P8, P10, P11, P12, P13, P14). Technology for younger generations could mean less social contact and interactions (benefit), whereas older generations may view this as an adverse effect of the implementation, and they might want and require younger generations to develop their soft skills.

External factors that might hinder the acceptance and adoption of technology include social factors, economic background, infrastructure, globalization and currency exchange. The CAs’ adoption and acceptance of 4IR technologies was influenced by the interplay of numerous external factors, namely, social factors (P1, P2, P4, P5, P6, P8, P10, P11, P12), economic background and infrastructure (P2, P5, P9, P12).

Social factors (P1, P4, P5), social exposure and interactions (P1, P2, P4, P8) played a significant role for the current study’s participants. This demonstrated how a person’s background and experience with the 4IR may affect their readiness to embrace and utilize the technologies associated with the 4IR. This resonated with previous literature. Social media is shaping CAs’ perception and awareness of technology as well as the rate of adoption and acceptance. P4 highlighted certain boundaries, and if she perceived that the social platform might penetrate the boundary, she would rather stay away – rejecting that platform. Previous literature also acknowledged the role of social influences and professional networks in technology acceptance (Grosu et al., 2023).

According to the participants (P2, P5, P9, P12), infrastructure and economic considerations could significantly impact how quickly technology is adopted. This was consistent with earlier research highlighting the influence of technological infrastructure and economic conditions on technology adoption rates (Davis, 2016; Schwab, 2016). Budnik et al. (2017) further provided examples of economic issues, explicitly mentioning power outages causing certain countries to be unable to access data or utilize certain technologies, affecting the pace of adoption.

Prior studies have well-documented client requirements for improved efficiency and cost-cutting while preserving quality (IFAC, 2017). The fact that client demands directly impact CAs’ decisions about which technologies to adopt highlights how crucial it is to meet client needs to be competitive and relevant in the industry. Additionally, the literature supported the impact of customers’ readiness for and comprehension of 4IR technologies on CAs’ adoption. It has been shown that clients who are hesitant or unprepared to deploy new technologies can make it difficult for CAs to effectively adopt and integrate these systems (P4, P7, P8, P12, P14). Previous research looked at the impact of client firms on the technological landscape of the accounting profession, and the interaction between client expectations and CAs’ adoption of technology has been highlighted (Philbeck and Davis, 2018).

## 6 Conclusion and recommendations

This study focuses on CAs perspectives of the 4IR and its implications for the profession. The premise for this study derives from the fact that the 4IR is profoundly restructuring several industries, including accounting, by redefining jobs, requiring new skills, and changing professional expectations. Understanding how accountants perceive and respond to these changes provides essential insights into managing the accounting profession’s metamorphosis in the face of technological breakthroughs in the 4IR.

The key conclusions of this study demonstrate the 4IR’s enormous impact on the CA profession. Notably, rapid task automation via technologies such as AI is posing a challenge to traditional CA roles, forcing a change towards more analytical thinking and strategic insight. Participants agreed that in order to effectively navigate the changing terrain, CAs must develop critical thinking abilities and data analysis ability. Furthermore, age dynamics play a role, with younger CAs being more comfortable and familiar with technology, while older generations may struggle to adapt to these changes. Despite fears about job loss due to technology, members were largely optimistic about the 4IR’s professional development potential.

The study’s merits stem from its comprehensive data collection approach, which includes semi-structured online interviews with a varied group of CAs. This strategy ensured the collection of many perspectives, which is critical for comprehending the complex effects of the 4IR on the CA profession. Thematic analysis enabled a systematic review of the data, which improved the reliability and credibility of the conclusions. Importantly, the study’s congruence with current literature and production of new insights add significantly to the body of information about the 4IR’s influence on accounting professionals.

### 6.1 Limitations of the study

Despite the important insights gained, numerous limitations must be noted. The study’s sample approach, which is mostly based on purposeful sampling, may induce biases and limit the representation of varied viewpoints within the CA community. Furthermore, the qualitative character of the study may contribute to subjectivity and social desirability biases in participants’ responses. The continually evolving nature of technology also presents a challenge, as conclusions may fail to adequately capture the significance of current improvements. Furthermore, the study’s emphasis on CA perspectives may neglect important insights from client perspectives, necessitating further research in this area. This is a qualitative research study with a small sample size and the authors do not claim any generalizability of the findings.

### 6.2 Recommendations for practice

To effectively navigate the 4IR, CAs should prioritise continuous learning and upskilling efforts, particularly those involving critical thinking and data analytics. Collaboration between CAs and professional bodies is vital for developing comprehensive training programmes that are in line with technological improvements. Furthermore, cultivating a culture of curiosity and openness to change inside CA organizations might help with smoother transitions during technology shocks. Professional bodies and policymakers should actively support CAs during the 4IR transition. This includes revising Continuing Professional Development (CPD) regulations to include technology-focused modules and encouraging digital literacy in the field through educational programmes. Future study directions should include long-term effect studies, an examination of regional and sectoral differences in education and training requirements, and an investigation of the role of professional bodies in CA technology adoption. These paths will help us gain a better grasp of the ongoing transformation in the CA profession as we enter the 4IR.

## Data Availability

The raw data supporting the conclusions of this article will be made available by the authors, without undue reservation.
